# Supplementary figures and images for: Comprehensive Single Cell Analyses of the Nutritional Environment of Intracellular Salmonella enterica
Source: Front Cell Infect Microbiol. 2021 Mar 23;11:624650. doi: 10.3389/fcimb.2021.624650 (PMC8021861; doi:10.3389/fcimb.2021.624650)

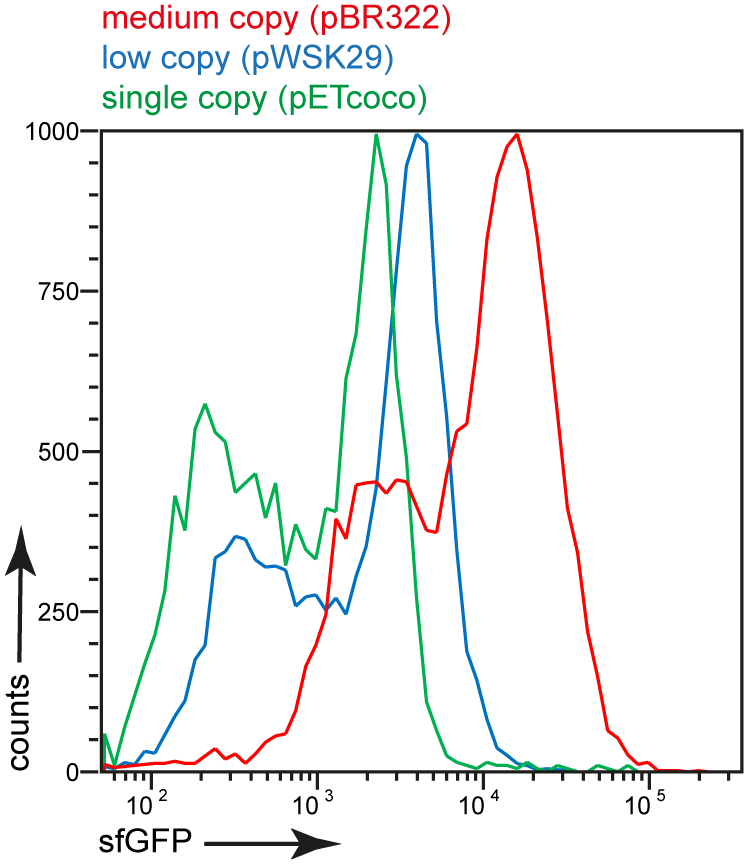

Supplement: Supplementary Figure 1 — Comparison of induction of PsitA::sfGFP in plasmids with different copy numbers. HeLa cells were infected at MOI 5 with STM WT containing iron reporters p5008, p5235 or p5564 harboring PsirA::sfGFP in plasmid backbones with various copy numbers. The cells were lysed and fixed 16 h p.i. Subsequently, the bacteria were subjected to FC to quantify the sfGFP intensity of the PsitA-induced bacteria. Data from one biological replicate. [file Image_1.tif]

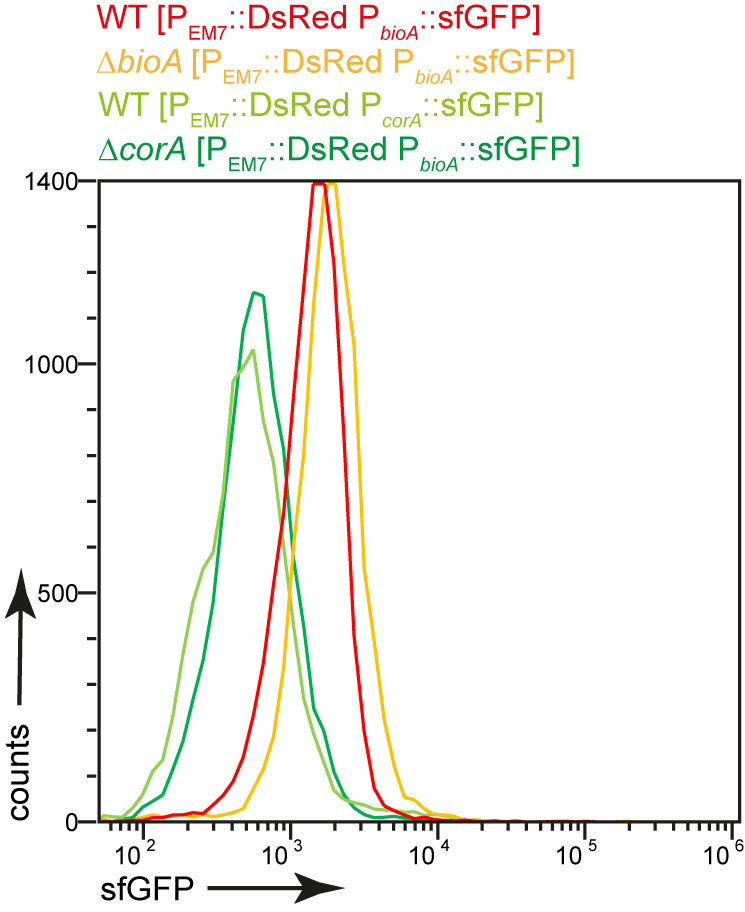

Supplement: Supplementary Figure 2 — Comparison of reporter induction in STM WT and mutant strains. HeLa cells were infected at MOI 5 with STM WT and mutant strains deficient in bioA or corA, containing the reporter p5067 or p5078 as indicated. The cells were lysed 16 h p.i., STM released, and fixed. Subsequently, the bacteria were subjected to FC to quantify the sfGFP intensity. Data from one biological replicate. [file Image_2.tif]

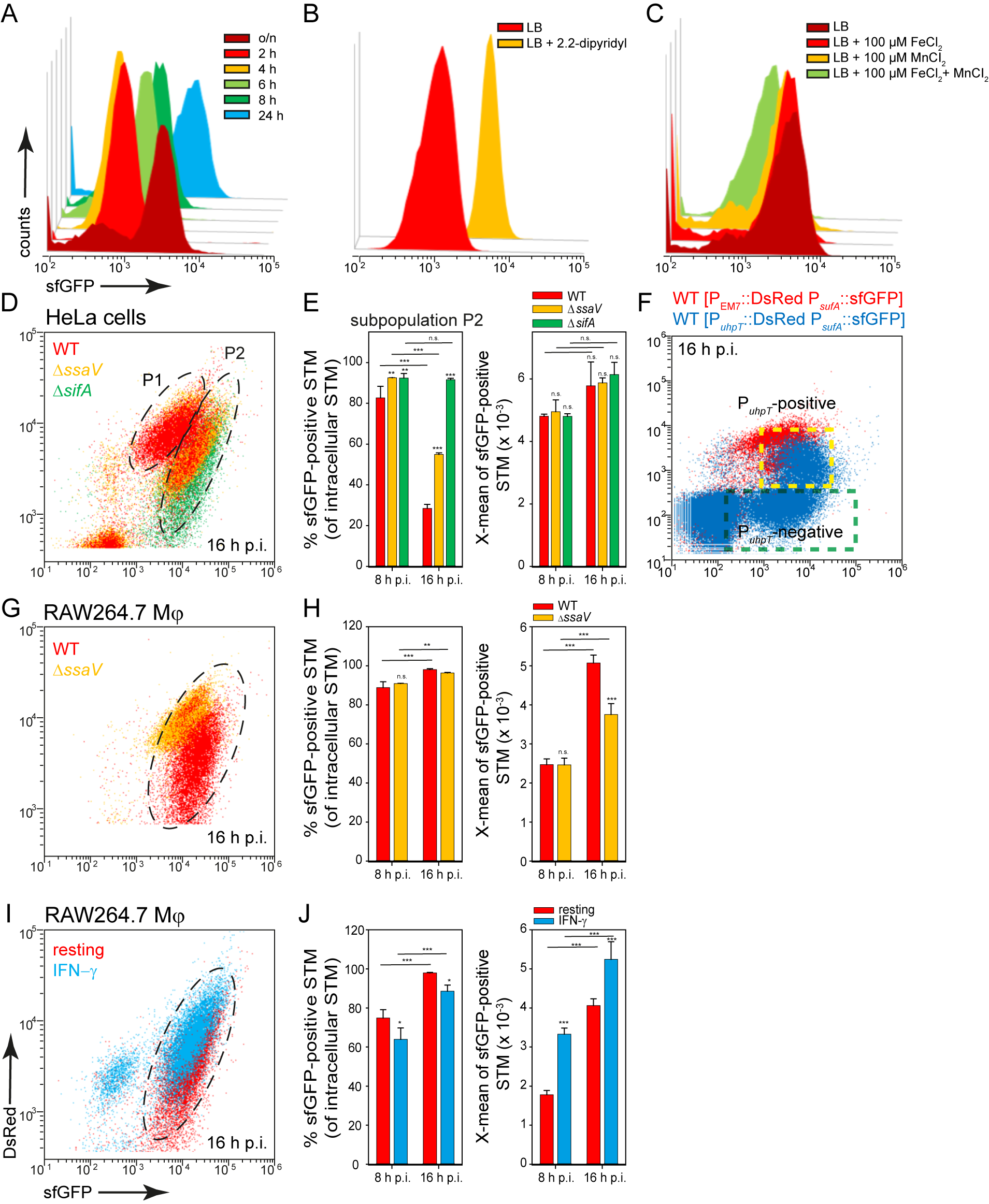

Supplement: Supplementary Figure 3 — An alternative dual fluorescence reporter for measuring iron limitation. STM harboring p5069 for constitutive expression of DsRed, and sfGFP under control of PsufAwas cultured in LB media. Induction of PsufA::sfGFP in vitro was determined by FC. (A) STM WT [p5069] was grown o/n in LB, diluted 1:31 in fresh LB and subcultured for various time points as indicated. Samples were collected as indicated. (B) WT [p5069] was grown o/n in LB, diluted 1:31 for 3.5 h in fresh LB without or with 200 µM 2.2-dipyridyl. (C) WT [p5069] was grown o/n in LB, or LB complemented with FeCl2 and/or MnCl2 as indicated. Samples were collected after 3.5 h of culture. sfGFP intensities of PsufA-positive bacteria of a representative experiment are shown, the value for the induction of the reporter was derived from at least 3 independent experiments. (D–J) Host cells were infected at MOI 5 with STM strains as indicated, each containing the iron reporter p5069. HeLa cells (D–F) and RAW macrophages (G-J) were analyzed as described for Figure 3 . Mean values and standard deviations from triplicates of a representative experiment are shown. Statistical analyses are indicated as for Figure 3 . (E) HeLa cells were infected with STM WT harboring p5190 [PuhpT::DsRed PsufA::sfGFP] (blue) and p5069 [PEM7::DsRed PsufA::sfGFP] (red). [file Image_3.tif]

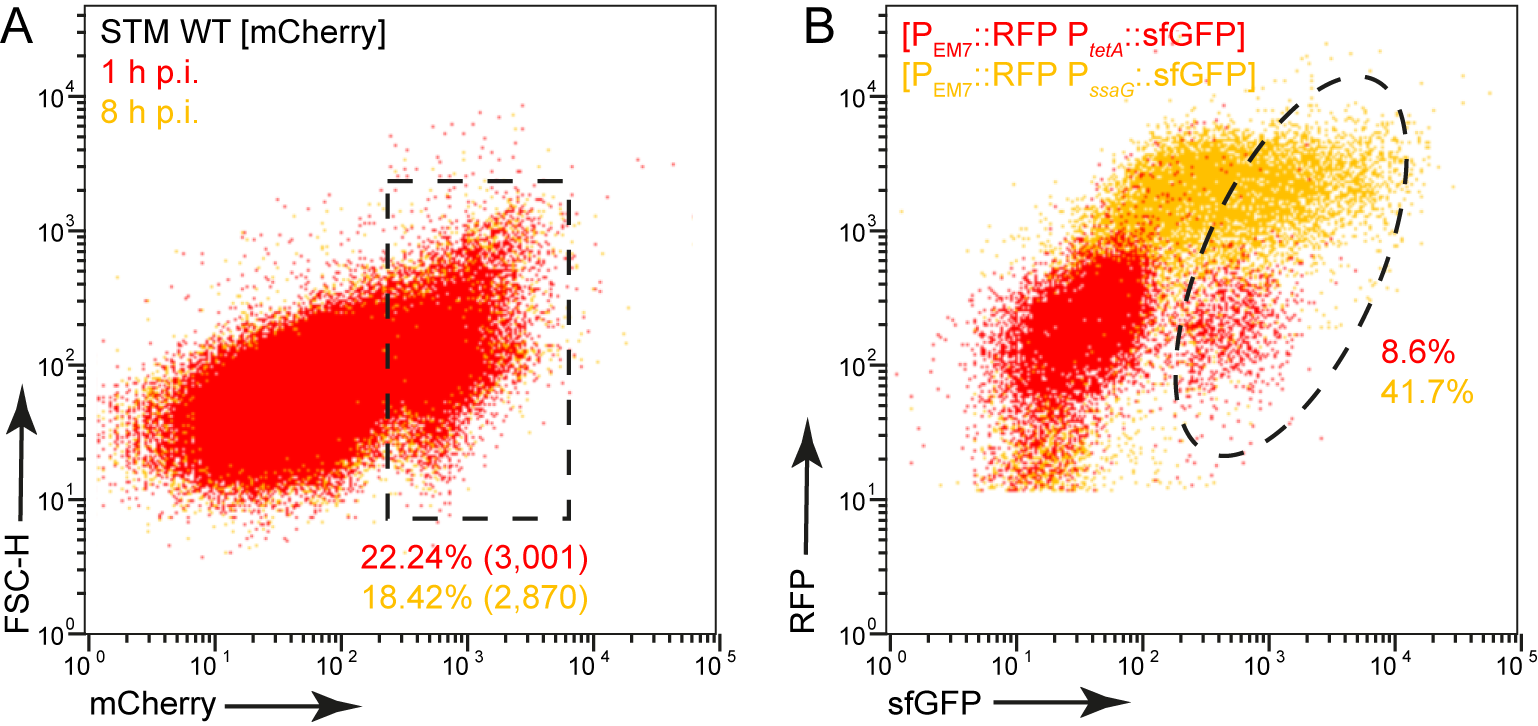

Supplement: Supplementary Figure 4 — Intracellular proliferation, SPI2 gene expression, and metabolic activity of STM in human macrophages. (A) Human macrophages were infected at MOI 5 with STM WT [mCherry] constitutively expressing mCherry. The cells were detached and fixed at 1 h (red) or 8 h p.i. (orange). Subsequently, at least 10,000 STM-infected macrophages per time point were analyzed by FC. (B) Human macrophages were infected at MOI 25 with STM WT [p4928] (red) or STM WT [p3776] (orange). For infection with STM [p4928], induction of the reporter was induced by addition of AHT to 100 ng x ml-1 at 6 h p.i., and cells were analyzed 8 h p.i. STM was released, fixed and subsequently subjected to FC to quantify the sfGFP-positive bacteria. Data from one biological replicate. [file Image_4.tif]
