# Supplementary material for: Comprehensive Single Cell Analyses of the Nutritional Environment of Intracellular Salmonella enterica
Source: Front Cell Infect Microbiol. 2021 Mar 23;11:624650. doi: 10.3389/fcimb.2021.624650 (PMC8021861; doi:10.3389/fcimb.2021.624650)
Supplement: Supplementary file 5 [file Table_1.docx]

**Table S1. Oligonucleotides used in this study**

| Designation | Sequence 5’ – 3’ |
| --- | --- |
| Vf-p4889 | ATGCGCAAAGGCGAAGAACTGTTTACCGGTGTGGTGCCGA |
| Vr-p4889 | GGCCGGCATCACCGGCGCCACAGGTGCGGTTG |
| 1f-P*sitA* | CAACCGCACCTGTGGCGCCGGTGATGCCGGCCTAGGCGAACAGCGCGTCGTA |
| 1r-P*sitA* | CACCGGTAAACAGTTCTTCGCCTTTGCGCATAGTATCCCTCGCAACAATGTGG |
| 1f-P*znuA* | CAACCGCACCTGTGGCGCCGGTGATGCCGGCCTTGCGGGACATAGCCGATA |
| 1r-P*znuA* | CCGGTAAACAGTTCTTCGCCTTTGCGCATAAATGCGACTAATCATCGTAAT |
| 1f-*sufA*-p4889 | CTGTGGCGCCGGTGATGCCGGCCAAATGGGGTTCTGACCTC |
| 1r-*sufA*-p4889 | CAGTTCTTCGCCTTTGCGCATAGAGTTACCTCGCGTTCT |
| 1f-*bioA*-p4889 | CTGTGGCGCCGGTGATGCCGGCCCTTCCAGCCCGGTTTTATAG |
| 1r-*bioA*-p4889 | CAGTTCTTCGCCTTTGCGCATAATCGACTTGTAAACCAAAT |
| 1f-*corA*-p4889 | CTGTGGCGCCGGTGATGCCGGCCTATTGCTTTTTCGCGTTTCGTTGCGT |
| 1r-*corA*-p4889 | CAGTTCTTCGCCTTTGCGCATGACCGGGACTCCCAATGCGT |
| 1f-*kdpA*-p4889 | CTGTGGCGCCGGTGATGCCGGCCTCCGACCACTTATTTTCACG |
| 1r-*kdpA*-p4889 | CAGTTCTTCGCCTTTGCGCATCAGAACGCCTCCGCATTA |
| Vr p4889 ex EM7 | ATCGATAGCTGGTCGACAACCAT |
| 1f p4507 P*uhpT* | TCGGCGTGGGTATGGTGGTGCCGGCCACGATGCGTC |
| 1r p4507 DsRed | TTGTCGACCAGCTATCGATGAAGCTTCTACAGGAACAGGTGGTG |
